# Accelerated Neuronal Cell Recovery from *Botulinum* Neurotoxin Intoxication by Targeted Ubiquitination

**DOI:** 10.1371/journal.pone.0020352

**Published:** 2011-05-24

**Authors:** Chueh-Ling Kuo, George A. Oyler, Charles B. Shoemaker

**Affiliations:** 1 Department of Biomedical Sciences, Tufts Cummings School of Veterinary Medicine, North Grafton, Massachusetts, United States of America; 2 Synaptic Research LLC, Baltimore, Maryland, United States of America; The University of Akron, United States of America

## Abstract

*Botulinum* neurotoxin (BoNT), a Category A biodefense agent, delivers a protease to motor neuron cytosol that cleaves one or more soluble NSF attachment protein receptors (SNARE) proteins involved in neurotransmission to cause a flaccid paralysis. No antidotes exist to reverse symptoms of BoNT intoxication so severely affected patients require artificial respiration with prolonged intensive care. Time to recovery depends on toxin serotype because the intraneuronal persistence of the seven known BoNT serotypes varies widely from days to many months. Our therapeutic antidote strategy is to develop ‘targeted F-box’ (TFB) agents that target the different intraneuronal BoNT proteases for accelerated degradation by the ubiquitin proteasome system (UPS), thus promoting rapid recovery from all serotypes. These agents consist of a camelid heavy chain-only V_H_ (VHH) domain specific for a BoNT protease fused to an F-box domain recognized by an intraneuronal E3-ligase. A fusion protein containing the 14 kDa anti-BoNT/A protease VHH, ALcB8, joined to a 15 kDa F-box domain region of TrCP (D5) was sufficient to cause increased ubiquitination and accelerate turnover of the targeted BoNT/A protease within neurons. Neuronal cells expressing this TFB, called D5-B8, were also substantially resistant to BoNT/A intoxication and recovered from intoxication at least 2.5 fold quicker than control neurons. Fusion of D5 to a VHH specific for BoNT/B protease (BLcB10) led to accelerated turnover of the targeted protease within neurons, thus demonstrating the modular nature of these therapeutic agents and suggesting that development of similar therapeutic agents specific to all botulinum serotypes should be readily achievable.

## Introduction

Botulism is caused by exposure to *Clostridium botulinum* neurotoxin (BoNT), a CDC Category A biodefense threat agent for which no antidote exists to reverse the symptoms of paralysis after onset. Intoxication is caused when the BoNT protease light chain (Lc) domain is delivered to the presynaptic terminal of motor neurons by the heavy chain (Hc) domain. In the presynaptic terminal the Lc cleaves SNARE proteins and inactivates neurotransmission [1], [2], [3], [4], [5], [6], [7], [8]. Seven different BoNT serotypes have been discovered to date (BoNT/A-G). The Lc proteases of the seven different BoNT serotypes have distinct active sites that cleave different sites in one or more SNARE proteins [3], [4], [9], [10], [11]. Thus, to protect against all known forms of BoNT, conventional small molecule drug development would need to be separately performed for each of the seven different drug targets, and perhaps even some of the subtypes. This challenge, together with other extreme hurdles confronting BoNT small molecule drug development, seriously complicates efforts to develop agents to treat botulism. New therapeutic paradigms are urgently needed to counter the enormous risks associated with these easy-to-obtain, easy-to-produce and extremely dangerous bioterror agents.

It is known that persistence of the symptoms of botulism varies dramatically following intoxication by different BoNT serotypes [12]. BoNT/A, the serotype with the longest persistence, has proven the most useful for therapeutic applications but also is considered the most dangerous as a biodefense threat. Persistence of symptoms has been related to prolonged survival of the Lc in the presynaptic terminal [13]. We reported evidence that this variation is due to the variable susceptibility of different BoNT Lcs to ubiquitination and proteasome-mediated turnover [14]. Furthermore, we showed that targeted ubiquitination of BoNT protease accelerated its turnover in neuroblastoma cells [14]. The biomolecules employed were large and not very specific for the BoNT protease and thus not practical for therapeutic use. Here we report development of biomolecules that are highly specific for BoNT proteases, small and stable enough to be practical for therapeutic use, and capable of accelerating BoNT protease turnover leading to a more rapid ‘molecular cure’ of intoxicated neurons.

Our therapeutic strategy builds on the demonstration by Zhou et al. [15] that a fusion protein of the F-box protein, β-TrCP, and an artificial protein binding domain can target a naturally stable protein for rapid proteasomal degradation. β-TrCP associates with Skp1 and Cullin to form the SCF complex, a multimeric E3 ubiquitin-ligase [16], [17] previously shown to be expressed in neuronal cells [18]. F-box proteins like β-TrCP contain two modular domains: a protein-protein interaction domain for binding substrates and the F-box which is required for association into the E3-ligase complex [19], [20]. Using this concept, we sought to engineer an artificial F-box protein containing a minimal F-box domain from β-TrCP and a small targeting domain that specifically binds to BoNT proteases.

The antigen binding V_H_ region of camelid heavy-chain-only antibodies, also called VHHs, were used as the BoNT LC protease targeting domain [21], [22]. VHHs are small, stable, well-expressed proteins that bind their target with high affinity and specificity, have excellent solubility properties, and often are potent inhibitors of target protein function [21], [23], [24], [25]. We previously reported the identification of high affinity VHHs (<10 nM K_D_) that recognize the proteases from either BoNT/A or BoNT/B, and demonstrated that these VHHs retain their binding properties within neuronal cell cytosol [26]. Here we show that fusions of these VHHs to a minimal F-box domain, called ‘targeted F-box’ (TFB) agents, effectively promote turnover of BoNT/A or BoNT/B proteases and accelerate neuronal recovery from symptoms of BoNT intoxication. Because of the modular nature of these antidotes, it should be straightforward to develop similar agents targeting all seven BoNT serotypes and subtypes by substituting the VHH with other VHHs having the appropriate specificity. Ideally, these TFB agents would be delivered to intoxicated neurons in botulism patients by a neuronally targeted delivery vehicle; for example as fusions to an atoxic mutant form of BoNT. If successful, such therapy would lead to shortened persistence of paralysis in botulism patients, thereby reducing the danger posed by these potential terror agents.

## Results

### Fusions of TrCP F-box to a BoNT Lc-specific VHH specifically reduce steady-state Lc expression levels within neuroblastoma cells

We previously demonstrated that the camelid heavy-chain-only V_H_ (VHH), ALcB8, binds to BoNT/A Lc protease (ALc) within neuronal cells and inhibits its protease activity [26]. The ALcB8 VHH was expressed as a fusion protein with the F-box protein, TrCP, to create a “targeted F-box” (TFB) designed to promote the specific, SCF E3-ligase mediated polyubiquitination of ALc and consequent proteasome-mediated degradation [15]. Initially, TFB function was measured indirectly through ALc activity since the exceedingly low level of ALc within intoxicated neuronal cells made it impractical to directly measure turnover. The B8-TrCP TFB fusion protein or ALcB8 alone were expressed within BoNT/A intoxicated neuroblastoma Neuro 2A (N2A) cells together with the ALc substrate, SNAP25, expressed as an indicator protein flanked by yellow fluorescent protein (YFP) and cyan fluorescent protein (CFP) [26]. Cells expressing B8-TrCP were reproducibly found to prevent cleavage of the co-transfected indicator protein, and more effectively than ALcB8 alone (Figure S1A). The B8-TrCP TFB was itself heavily polyubiquitinated in N2A cells and its steady state expression level was thus very low (Figure S1B), especially compared to ALcB8. This indicated that the ability of B8-TrCP to reduce ALc activity in intoxicated cells was due to accelerated turnover rather than protease inhibition.

**Figure 1:**
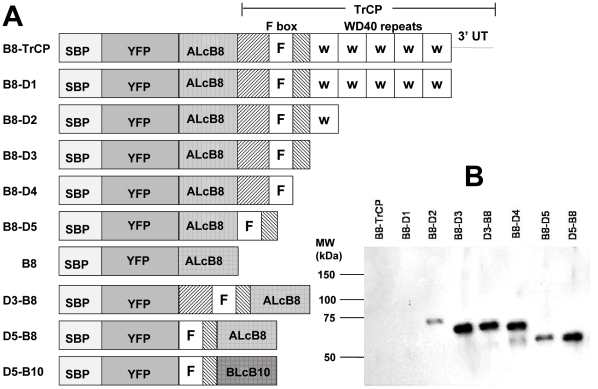
Expression of Lc-targeting TFBs containing various truncations of TrCP cDNA. (A) Schematic of various expressed TFB fusion proteins containing different regions of TrCP cDNA including the F-box domain (scale approximate). All fusion proteins contain a streptavidin binding peptide (SBP) and a yellow fluorescent protein (YFP) domain at the amino end. Each TFB contains the BoNT/A Lc binding VHH, ALcB8, or the BoNT/B Lc binding VHH, BLcB10. Some proteins contain one or more WD40 repeat (w). (B) Western blot of recombinant TFBs expressed in M17 cells. M17 cells were transfected with expression plasmids for the different ALcB8 TFB protein diagrammed in A. The proteins were affinity purified with GST-ALc, resolved by SDS-PAGE and detected by Western blot with anti-GFP antibody (Santa Cruz). Data shown is representative of three separate experiments. The 117 kDa B8-TrCP and B8-D1 proteins were apparent on longer exposures.

The F-box domain within TrCP that is required for association with Skp1 within the SCF E3-ligase complex [20] is only about 50 amino acids. A series of expression vectors (represented in Figure 1A) were prepared to identify the minimum portion of TrCP required to retain TFB function. Steady-state expression levels of the B8-TrCP fusion protein were not much improved by removal of the TrCP 3′ untranslated region (UTR) alone (B8-D1) or most of the TrCP WD40 repeats (B8-D2) (Figure 1B). Removal of all TrCP WD40 repeats (B8-D3), though, resulted in much higher steady-state expression of the TFB and also shifted the predominant sub-cellular localization from the typical TrCP nuclear site to the cytosol as previously observed [27], [28] (Figure S2). Deletions of additional regions of TrCP flanking the F-box domain (B8-D4, B8-D5) also displayed improved steady-state expression levels (Figure 1B) and cytosol localization (Figure S2). Swapping the VHH and F-box domains (D3-B8, D5-B8, Figure 1A) did not significantly alter expression levels or localization. Expression of all TFBs in which the ALcB8 was fused to the TrCP F-box region present in D5 (aa 175–293) protected N2A cells from BoNT/A cleavage of SNAP25 (Figure S3). A further truncation of TrCP (aa 175–233) produced variable results and was not pursued.

A second TFB was produced in which the ALcB8 VHH targeting domain was replaced with a VHH (BLcB10) having specificity for BoNT/B Lc (BLc) [26]. The TrCP F-box domain, D5, was fused in frame with BLcB10 in the orientation in which the VHH is at the carboxyl terminus (D5-B10, Figure 1A). The D5-B10 protein was expressed in the neuroblastoma cell line, M17, and shown to retain the ability to bind BLc in cells based on pull-down assays (Figure S4).

**Figure 2:**
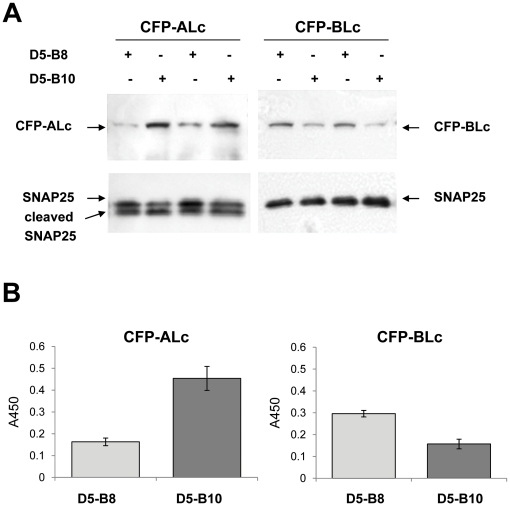
BoNT Lc targeted TFBs reduce the steady-state level of co-expressed Lc in transfected neuroblastoma cells. N2A cells were co-transfected with expression vectors for CFP-ALc or CFP-BLc and for TFB D5-B8 or D5-B10 as indicated. (A) Western blots. 24 hrs post-transfection, cell extracts were prepared and resolved by SDS-PAGE. CFP-Lc and SNAP25 expression levels were detected by Western blotting using anti-GFP or anti-SNAP25 antibody. (B) Capture ELISA. Cell extracts prepared from transfected cells in A were quantified by capture ELISA. Background absorbance was subtracted from the absorbance at OD450 nM. Data are presented as averages ± standard deviation calculated from three independent samples and compared by unpaired t test. The differences between each pairing are highly significant (p<0.001).

The ALc-specific TFB, D5-B8, and the BLc-specific TFB, D5-B10, were co-expressed within N2A cells together with ALc and/or BLc and tested for their influence on Lc steady-state levels. Expression of D5-B8 in N2A cells reduced steady-state levels of ALc compared to cells expressing D5-B10 (Figure 2A). In contrast, levels of BLc were lower in N2A cells co-expressing D5-B10 than those expressing D5-B8. The protein levels for ALc were quantified by capture ELISA and shown to be reduced about 65% in N2A cells that co-expressed the ALc TFB, D5-B8, as compared to D5-B10 (Figure 2B). N2A cells expressing the BLc TFB, D5-B10, contained about 50% of the BLc level found in cells expressing D5-B8 (Figure 2B). Similar results were obtained when both ALc and BLc were expressed in the same N2A cells expressing either D5-B8 or D5-B10 (Figure S5). SNAP25 served as the loading control in these experiments. These results demonstrate the modular nature of the TFBs in which the VHH domains can be exchanged with other VHHs having a different specificity and thereby target a different protein for accelerated degradation.

### TFBs promote target-specific ubiquitination

**Figure 3:**
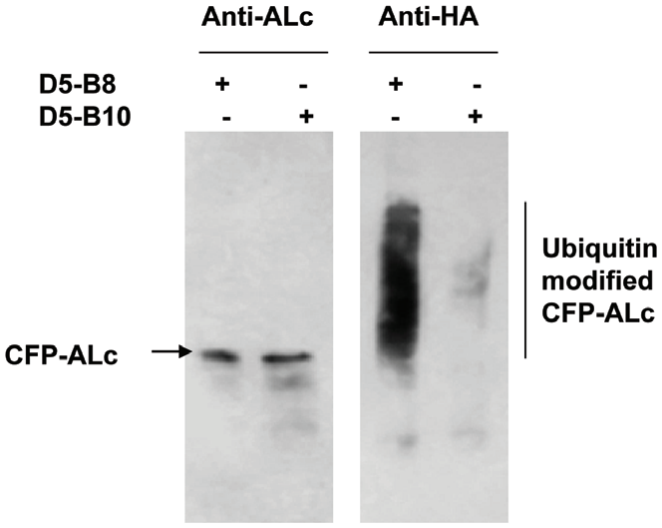
TFBs promote target-specific polyubiquitination. M17 cells were co-transfected with HA-ubiquitin, CFP-ALc and expression plasmids for ALc-specific TFB (D5-B8) or BLc-specific TFB (D5-B10) as indicated. 24 hrs post-transfection, cells were treated with 10 µM of MG132 for 6 hrs and cell lysates were prepared. Purified recombinant GST-ALcB8 was added to the cell extracts and CFP-ALc was purified by glutathione affinity. Eluted protein was resolved by SDS-PAGE. CFP-Lc expression levels and HA-ubiquitin modification were detected by Western blotting using sheep anti-ALc antibody or anti-HA antibody and the results shown are representative of four separate experiments.

CFP-ALc was co-expressed with the TFBs D5-B8 or D5-B10 in the presence of the proteasome inhibitor, MG132, to permit accumulation of polyubiquitinated proteins. The cells were also co-transfected with an expression plasmid for HA-ubiquitin. The CFP-ALc was purified from cell extracts by affinity to GST-ALcB8. The use of the VHH ALcB8 to purify the ALc should eliminate (by competition) co-purification of any D5-B8 that remained bound to the CFP-ALc in the extract and which could lead to contaminating polyubiquitinated protein. The purified ALc from each extract was analyzed by Western blot (Figure 3) and shown to contain CFP-ALc. The amount of extract loaded was normalized such that the CFP-ALc levels were nearly the same. When an identical Western blot was analyzed for HA, it became clear that the ALc co-expressed with D5-B8 was much more heavily ubiquitinated than ALc co-expressed with D5-B10. These results show that the TFB D5-B8 is promoting ubiquitination of ALc within the transfected M17 cells.

### TFBs accelerate protein turnover in a target-specific manner

**Figure 4:**
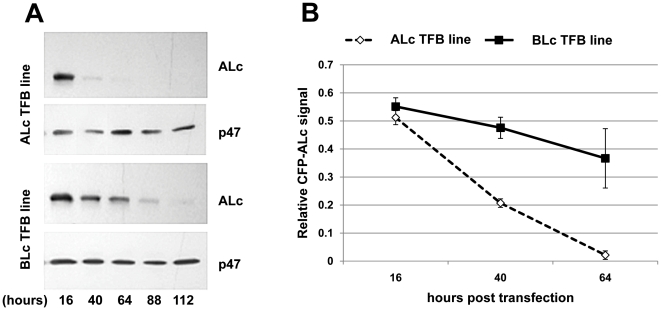
ALc turnover is accelerated in the presence of TFB D5-B8. (A) ALc expression detected at various times post-transfection in cell lines expressing TFBs. Expression plasmid for CFP-ALc was transfected into cells stably expressing D5-B8 (ALc TFB) or D5-B10 (BLc TFB). Cell lysates were prepared at the indicated time points and resolved by SDS-PAGE. The expression level of CFP-ALc and p47 were monitored by Western blotting using anti-ALc Ab or anti-p47 Ab for detection. (B) Quantitative analyses of ALc expression levels based on scanning densitometry. Western blots such as shown in A were scanned and the signals relative to an internal standard (p47) were calculated and plotted. Data are presented as the average of three sample points ± standard deviation and compared by two-way analysis of variance (ANOVA). The differences between the two cell lines are highly significant (p<0.005) Similar results were obtained in three separate experiments and also using SNAP25 as the internal standard.

M17 stable cell lines that express a transgene for either the TFB D5-B8 or D5-B10 were created by lentivirus vector transduction. Virtually all cells in these populations express the TFB transgene based on YFP fluorescence. The D5-B8 and D5-B10 cell lines were transfected with an expression plasmid for CFP-ALc and the level of ALc expression (and p47 as a loading control) was detected at various times post-transfection by Western blot (Figure 4A) and quantified by scanning (Figure 4B). The apparent half life for CFP-ALc when co-expressed with D5-B8 (ALc TFB line) was ∼1.5 days while in cells co-expressing D5-B10 (BLc TFB line), the half life of CFP-ALc was ∼3.7 days. The studies clearly show that D5-B8 accelerated the turnover of CFP-ALc (p<0.005). In a separate experiment, the levels of ALc, TFB and SNAP25 were each individually assessed by Western blot at days 3, 4 and 5 (Figure S6). Once again, the ALc levels were reduced much more rapidly when co-expressed with D5-B8 compared to D5-B10 while the levels of the stably expressed TFB and endogenous SNAP25 remained nearly constant as expected. The efficacy of TFBs to accelerate Lc turnover was dependent on proteasome function as no differences in ALc and BLc steady-state levels were observed in D5-B8 or D5-B10 cells treated with the proteasome inhibitor, MG132 (Figure S7).

M17 cells that stably express the TFB D5-B8 or D5-B10 were compared for their susceptibility to BoNT/A intoxication as assessed by cleavage of SNAP25. The D5-B8 cells were found to become intoxicated to a significantly lesser degree (<30% SNAP25 cleavage) than D5-B10 cells (Figure S8) or parental M17 cells (70–80% SNAP25 cleavage) (p<0.005). As the VHH ALcB8 component of D5-B8 is a potent inhibitor of ALc protease [26], it is not possible to separate the contributions of protease inhibition and accelerated ALc turnover in this assay.

### ALc TFB (D5-B8) promotes accelerated recovery of M17 cells following BoNT/A intoxication

**Figure 5:**
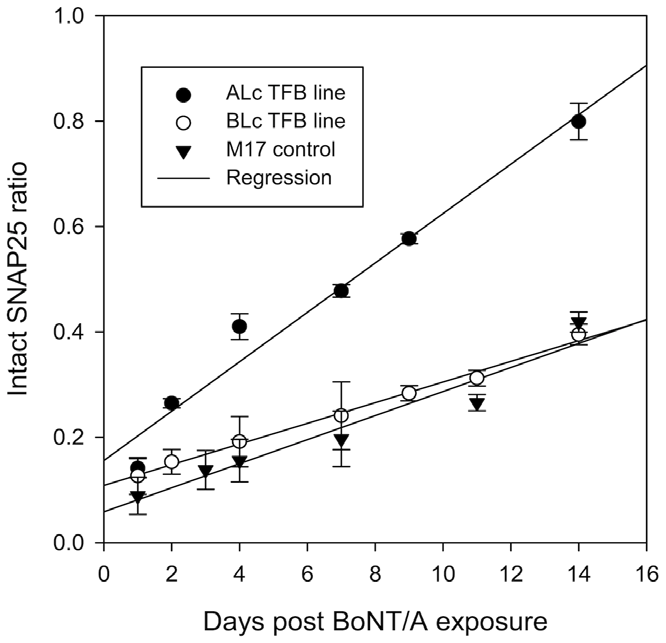
Intact endogenous SNAP25 levels recover more rapidly following BoNT/A intoxication in cells expressing ALc TFB (D5-B8). Control M17 cells or M17 cells stably expressing ALc TFB D5-B8 or BLc TFB D5-B10 were exposed to 10 nM BoNT/A for 24 hrs. Cell lysates were prepared at indicated time points, resolved by SDS-PAGE and Western blots were performed to detect SNAP25 as shown in Figure S9. The fraction of intact SNAP25 was estimated by scanning densitometry. Data are presented as the average ± standard deviation and compared by two-way ANOVA. The differences between ALc TFB line and BLc TFB line, and between ALc TFB line and M17 control, are highly significant (p<0.001). Data shown are representative of 10 separate experiments.

Finally we tested whether the levels of intact SNAP25 recover more rapidly following BoNT/A intoxication of neuroblastoma cells when expressing the ALc-targeting TFB, D5-B8. M17 cell lines that constitutively express either TFB D5-B8 or D5-B10 were intoxicated with BoNT/A. Cells were nearly confluent at the time of intoxication to limit new cell division that might dilute the intoxication effect. At various times post-intoxication, cells were harvested and assessed for the proportion of intact SNAP25 (Figure S9). With time, the proportion of intact SNAP25 recovered to some extent in all cases. In cells expressing D5-B8, intact SNAP25 recovered to near pre-intoxication levels in two weeks (Figure 5). Within control M17 cells or M17 cells expressing TFB D5-B10, intact SNAP25 represented less than 50% of the total SNAP25 after two weeks. In these experiments, the presence of D5-B8 promoted about 2.5 fold more rapid recovery vs. both controls and the difference was highly significant (p<0.001).

## Discussion

The therapeutic challenges of *Botulinum* neurotoxin poisoning are largely due to its extreme potency and the long persistence of the resulting flaccid paralysis. Currently there is no antidote for the symptoms of botulism once paralysis has become established. Development of small molecule drugs that inhibit BoNT proteases to reverse botulism is feasible, but faces enormous challenges. For example, at least seven BoNT serotypes exist, each having a protease with different substrate specificity, thus each requiring an independent drug development effort. Secondly, the protease must be continuously and completely inhibited for as long as it remains in the intoxicated neurons or a recurrence of symptoms will occur. Thirdly, clinical trials for botulism antidote drugs will be extremely limited and thus efficacy and toxicity from long exposure of the drugs will remain uncertain. Therefore, it is important also to seek alternative botulism therapies that reduce the persistence of the BoNT proteases within neurons leading to more rapid recovery from paralysis.

In this study, we successfully tested the novel concept of ‘VHH-targeted F-box’ (TFB) agents to promote the accelerated, target-specific, turnover of an intracellular protein. Specifically we demonstrated that a VHH specific for a *Botulinum* neurotoxin (BoNT) protease (Lc) and fused to the F-box domain from TrCP will promote polyubiquitination of the Lc in neuronal cell cytosol and accelerate its proteasome-dependent turnover. Furthermore, we demonstrate that TFBs retain their activity when the VHH (14 kDa) is fused to a 15 kDa region of β-TrCP containing the F-box domain. The modular nature of TFBs was demonstrated by replacing the ALc-specific VHH domain with a VHH targeting the protease of another BoNT serotype (BLc) and showing that this TFB promoted intracellular turnover of BLc, not ALc. The potential therapeutic application of TFBs was demonstrated by showing that neuronal cells intoxicated by BoNT/A recovered from a measurable symptom of intoxication (SNAP25 cleavage) at a significantly faster rate when the intoxicated cell expressed the appropriately targeted TFB.

This work builds on the seminal work of Zhou et al. [15] that used a fusion between β-TrCP and a protein with affinity for the normally stable retinoblastoma protein (pRB) to promote more rapid turnover of pRB. A related strategy called Protac (Proteolysis Targeting Chimeric Molecule) [29], [30] employs small molecules and peptides to act as a bridge between the SCF ubiquitin ligase and protein targets leading to ubiquitination and degradation of the target. These approaches require the identification or availability of a targeting domain having sufficiently high affinity and specificity to promote therapeutically useful target turnover. Our strategy employs VHHs as the targeting domain [31]. These proteins derive from the V_H_ domain of heavy-chain-only IgGs produced by camelid animals. VHHs are small, highly stable, single-domain binding agents that can be rapidly generated against virtually any target by a variety of approaches [32] including iterative, high-throughput methods using non-immune libraries [33]. The demonstration that VHHs are effective as the targeting domains thus opens the possibility of rapidly developing agents that target the accelerated turnover of all seven BoNT serotypes and, indeed, most any cytosolic target within cells.

The TFB with an ALc targeting VHH fused to a full-size TrCP proved exceedingly unstable and mostly localized to the nucleus so an effort was made to use truncated TrCP domains that may have improved expression and stability as well as a mostly cytosolic localization. We hypothesized that truncation of TrCP outside of the F-box would eliminate potential auto-ubiquitination sites and nuclear localization signals, and would lose normal activities which may prove harmful in a therapeutic context. We found that TFBs with substantial TrCP truncations protected N2A cells from BoNT/A-mediated cleavage of SNAP25 and were expressed in the cytosol to much higher steady-state levels than TFBs with full-size TrCP.

To directly test the TFBs for the ability to promote polyubiquitination and proteasome-mediated degradation of the targeted BoNT proteases, neuroblastoma cells were co-transfected with vectors driving co-expression of the TFBs and the BoNT Lc proteases. The presence of the ALc-targeting TFB, D5-B8, was shown to promote much enhanced polyubiquitination of ALc compared to all controls. More importantly, co-expression of ALc and the ALc TFB, D5-B8, led to significantly reduced steady-state levels of ALc. Co-expression of the BLc TFB, D5–D10, led to significantly reduced levels of co-transfected BLc. The two TFBs provided ideal negative controls for each other in these studies. The results also validated the modular concept of the TFBs in which the target specificity can be altered by swapping the VHH targeting domain.

To quantify the rate at which ALc is degraded within neuroblastoma cells expressing TFB D5-B8 or D5-B10, stable M17 cell lines were generated that constitutively express either the ALc TFB, D5-B8, or TFB D5-B10. Use of these cells allows a constant level of the TFB to be expressed throughout the experiment and thus eliminates background from cells that do not express TFB such as occurs with transient plasmid transfection. Following transfection of these TFB cell lines with an expression vector for CFP-ALc, levels of ALc were measured by Western blot and quantified by capture ELISA. ALc turnover was measured to be about 2.5 fold more rapid in the presence of D5-B8 as compared to D5-B10. This is a minimal estimate of the difference in turnover rates because it doesn't account for the continued, decreasing synthesis of ALc from the transgene which deflates turnover estimates especially during the early time points. The M17 cell line constitutively expressing D5-B8 was more refractory than control cells to BoNT/A intoxication based on cleavage of endogenous SNAP25. This indicates that much of the ALc entering cells during intoxication becomes inhibited and/or degraded by the presence of the TFB D5-B8. The fact that a low level of intoxication still occurs despite the presence of D5-B8 may indicate that ALc is partially sequestered from the cytosolic TFB during the intoxication process and the protease gains access to the membrane-associated SNAP25 before it can be bound by the TFB.

Finally we tested whether the presence of an appropriately targeted TFB could accelerate the recovery of neuronal cells from BoNT/A intoxication using SNAP25 integrity as the measure. Since the BoNT/A protease is eliminated from intoxicated neurons more rapidly in the presence of ALc-targeted TFB, the intact SNAP25 should also be renewed more rapidly. Studies demonstrated this to occur as M17 cells expressing D5-B8 recovered levels of intact SNAP25 at a significantly more rapid rate than controls. We speculate that the TFB D5-B8 is leading to more rapid molecular ‘cure’ of the neuron through elimination of the ALc, thereby permitting the neuron to remove cleaved SNAP25 and renew intact SNAP25 by normal metabolism.

The studies with TrCP truncation demonstrated that much of β-TrCP outside of the F-box domain was expendable for TFB function. This is consistent with the dogma that it is the 50 amino acid F-box domain that interacts with the SCF E3-ligase to recruit associated proteins for polyubiquitination [19]. Our results confirm that other regions of β-TrCP are not necessary for the recruitment of bound proteins for accelerated turnover. We also found that the orientation of the F-box relative to the VHH did not appear to influence the TFB function. In sum, the results indicate that precise positioning and spacing of the recruited target protein as it is bound to the F-box domain does not appear to significantly influence the availability of the target protein as a substrate for the E3-ligase.

The TFBs in this study were expressed with an amino terminal YFP partner to permit the monitoring of expression by fluorescence microscopy and to facilitate comparable detection of the many TFBs tested in Western blots using anti-YFP antibodies. The proteins also contained a small streptavidin binding peptide to permit pull-down analysis using streptavidin beads. While unlikely, it is possible that these fusion partners may have some influence on the function or stability of TFBs. Once a vehicle has been identified for the in vivo delivery of TFBs to intoxicated neurons, further development will likely be required to select a TFB agent having optimal therapeutic properties.

Our results indicate that TFBs have therapeutic potential as antidotes for botulism to promote the accelerated removal of protease from intoxicated neurons and to promote more rapid neuronal recovery. Clearly a significant challenge to the success of such therapy will be identifying a strategy for the delivery of TFB biomolecules to intoxicated neurons within patients. The strategy must deliver an effective number of molecules to a sufficient number of intoxicated neurons to reverse paralysis. Because the amount of protease within intoxicated neurons is very low and the TFBs may be able to catalytically promote destruction of multiple BoNT proteases, it is expected that the number of TFB molecules that must be delivered for efficacy is small. The ideal delivery system should be highly efficient and specific to neurons to minimize both the dose of agent required and the potential for side effects from non-specific delivery. Such a delivery system may be found in *Clostridial* toxins themselves which are highly evolved for efficient delivery of biomolecules to cells. Perhaps most promising would be BoNT itself as atoxic versions of the holotoxin have been created and produced in quantity [34] and these should be highly specific for neurons. Also it is has been shown that a BoNT delivery vehicle should be able to enter previously intoxicated neurons [35]. Furthermore, the BoNT heavy chain alone was recently shown to be an effective vehicle for the delivery of GFP into cells [36]. Other Clostridial toxin-based delivery systems, such as using *Clostridium difficile* toxin B (TcdB) [37], [38] or Clostridial C2 toxin [39], may have potential if they can be engineered to have neuronal specificity. The small size of TFBs at less than 30 kDa makes these biomolecules good candidates for delivery by *Clostridial* toxin-based vehicles as this is smaller than the natural cargo delivered by these systems.

## Materials and Methods

### Biosafety

All protocols were approved by the Tufts University Institutional Biosafety Committee and carried out under the CDC Select Agent Program following all applicable federal guidelines.

### Reagents

Antibodies used were: mouse anti-HA antibody (Sigma); rabbit anti-SNAP25 antibody (Sigma); goat anti-rabbit HRP antiserum (Sigma); goat anti-mouse HRP antibody (Santa Cruz); rabbit anti-GFP antibody (Santa Cruz). Sheep anti-BoNT/A Lc antiserum was a gift from Dr. Jean Mukherjee (Tufts University). Rabbit anti p47 was a gift of Dr. Hemmo Meyer (ETH, Zurich). Reagents for Western blotting, including Wash Solution and LumiGLO Chemiluminescent Substrate, tetramethylbenzidine (TMB) were purchased from KPL.

### Expression plasmids

VHH and β-TrCP coding DNAs were amplified by PCR and ligated into the mammalian expression vector, pcDNA3.1 (Invitrogen) fused in frame to an amino terminal yellow fluorescent protein (YFP) coding region. The expression plasmids also contain coding DNA for a streptavidin binding peptide (SBP) [40] at the amino terminus, upstream of the YFP. The complete β-TrCP cDNA (GenBank CAH70020) encodes 605 amino acids and includes the 3′ UTR. Truncated forms of β-TrCP all lacked the 3′ UTR and contained DNA encoding the following amino acids: D1, aa 1–605; D2, aa 1–379; D3, aa 1–293; D4, 1–233; D5, aa 175–293; D6, aa 175–233. ALcB8 and BLcB10 VHH coding sequences were the same as previously reported [26].

The BoNT/A (subtype A1) protease (ALc) expression plasmid contained ALc coding DNA within pcDNA3.1 fused to an amino terminal CFP domain (CFP/ALc) as described previously [26]. The BoNT/B protease (BLc) expression vector was prepared exactly as for ALc except that the ALc coding DNA was replaced with DNA encoding BLc, amino acids 1–440 from BoNT/B holotoxin. The expression plasmid for YFP/SNAP25/CFP indicator protein was described previously [26].

### Neuroblastoma cells

M17 (ATCC# CRL-2267) cells were maintained in Dulbecco's Modified Eagle Medium (DMEM) (Gibco) containing 10% fetal bovine serum (FBS) (Gibco). Neuro2a (ATCC# CCL-131) (abbreviated as N2A) cells were cultured in Minimum Essential Medium Eagle (MEME) (Gibco) plus 10% FBS. 2×10^5^ cells were seeded into wells of a 24-well plate and maintained at 37°C.

### Cell transfection and extract preparation

After 24 hrs, neuroblastoma M17 or N2A culture medium was replaced with fresh medium before experimental treatments. Cells at 80% confluence were used for transfection. For each well of a 24-well plate, 0.5 µg of plasmid was mixed into 50 µl of serum-free medium. Transfection reagent FuGene HD (Roche) was added into the plasmid mixture at a ratio of 1∶3 (DNA [µg]∶ Fugene [µl]) and incubated at room temperature for 15 min before the transfection mixture was applied to cells for 24 hrs. After transfection, cells were collected following trypsin treatment and washed once with 0.5 ml of Dulbecco s Phosphate Buffered Saline (DPBS) for cell extract preparation. For Western blotting analysis, total lysates were made by collecting cells in 50 µl of sample buffer [62.5 mM Tris-HCl, pH 6.8, 2% SDS, 10% glycerol and 0.002% bromophenol blue plus 5% beta-mercaptoethanol] and boiling for 10 min. For other applications, protein extracts were made by collecting cells in 50 µl of lysis buffer [DPBS containing 1× protease inhibitors, 1 mg/ml BSA, 0.1% Triton-X100] and incubated on ice for 30 min. Cell debris and protein extract were separated by centrifugation at 13,000 rpm for 15 min at 4°C.

### Streptavidin and glutathione affinity purification

25 µl of streptavidin beads (Dynabeads® M-280 Streptavidin, Invitrogen) were washed twice with 50 µl of DPBS and once with 50 µl of lysis buffer. Washed streptavidin beads were resuspended with 50 µl of cleared protein extract and incubated at 4°C for 16 hrs with rotation. Protein-bead complexes were washed 4 times with 100 µl of ice cold DPBS. Bound proteins were eluted from beads by adding 25 µl of sample buffer to the protein-bead complex and boiling for 5 min prior to gel electrophoresis. For glutathione affinity purification, 2 µl of glutathione magnetic beads from MagneGST™ Protein Purification System (Promega) were pre-coupled with 1 µg of recombinant GST fusion proteins by incubation at 4°C for 3 hrs. After coupling, the complex was used to co-purify proteins having affinity to the GST proteins from cleared cell lysates as above for Dynabeads beads. Protein eluates in sample buffer were resolved through SDS-PAGE (4–15% gradient gel, 8.6×6.8 cm (W×L), BioRad).

### BoNT/A intoxication and transduction

M17 cells were intoxicated with BoNT/A as previously described [41]. Briefly, a 50 µl solution of serum-free DMEM was prepared containing 0.75 µg of BoNT/A. FuGene HD (or DMEM control) was then added at the ratio (BoNT [µg]∶ FuGene HD [µl]) of 1∶3 and the mixture was incubated at room temperature for 15 min. The BoNT/A mixtures were then applied to cultured cells containing 0.5 ml fresh culture medium in a well of a 24-well plate to a BoNT/A final concentration of 10 nM. Following various incubation times, cell pellets were collected and dissolved in 50 µl of sample buffer and boiled for 10 min prior to gel electrophoresis. To measure recovery of endogenous, uncleaved SNAP25, 60 mm plates of M17 cells were intoxicated with 10 nM BoNT/A for 24 hrs. Intoxicated cells were washed twice with 5 ml of DPBS and split into 24 well plates. Cell extracts were collected at various times post-intoxication and processed as above.

### Capture ELISA

0.5 µg of an ALc-binding VHH, JDA-D12 (GenBank accession HQ700702), was coated onto each well of a 96 well plate at 4°C overnight for ALc capture. Streptavidin coated plates-high sensitivity (Thermo Scientific) were used for BLc capture. Cell extracts prepared from transfected cells were applied onto 5% skim milk/PBST (0.05% Tween-20) blocked plates and incubated at 4°C overnight. Captured Lc was detected with BoNT serotype-specific anti-Lc antisera followed by appropriate HRP-conjugated secondary antibodies. Signals were developed by adding TMB substrate and the absorbance was recorded at 450 nM with a Synergy™ HT Multi-Mode Microplate Reader.

### Generation of lentiviral vectors

Construction of lentiviral vectors carrying YFP-TrCP-D5/B8 and YFP-TrCP-D5/B10 coding DNA was accomplished by first cloning each respective PCR amplified coding region into the BamHI and XhoI sites of the transducing plasmid pLenti6.3/V5-DEST (Invitrogen). Viral particles were subsequently produced by co-transfecting 3.5 µg of the transducing plasmid with 7.1 µg HIV-1 gag-pol helper construct (Synaptic Research), and 2.8 µg of VSV-G expression plasmid (Synaptic Research) onto 80–90% confluent 293FT cells (Invitrogen) cultured in 100 mm plates. Culture medium that contained the budded viral vectors was collected 48 hrs after transfection and cleared of cell debris by centrifugation at 2,000 RPM for 10 minutes at 4°C (Sorvall RT 600D). The cleared viral supernatant was further concentrated by ultracentrifugation at 25,000 RPM for 90 minutes at 4°C (Beckman Coulter Optima™ XL-100K). Lastly, the viral vector pellet was soaked in 50 µl (1/200 the original volume) culture medium overnight, resuspended, and stored at −85°C until needed for transduction.

### Production of lentivirus transduced neuronal cell lines

M17 cells were seeded onto 12-well plates the day before transduction. On the day of transduction, lentiviral stock was thawed and diluted to different extents into 350 µl fresh complete medium. The original culture medium was removed from the cells and the fresh medium containing virus was then applied to the cells. Hexadimethrine bromide (Sigma) was added to the cells to a final concentration of 8 µg/ml. At 6 hrs post-transduction, cells were covered with enough complete medium for overnight incubation. The transduction procedures were repeated on the second day and culture medium was replaced with fresh, complete medium containing 5 µg/ml of blasticidin to select for stably transduced cells 24 hrs after the second transduction. Medium with blasticidin was replaced every 3–4 days until only fluorescent cells remained.

## Supporting Information

Figure S1 **TFB ALcB8-TrCP expressed in M17 cells inhibits BoNT/A cleavage of a SNAP25 indicator protein and is rapidly turned over by a proteasome-mediated process.** (A) Western blots of SNAP25 indicator protein levels +/− BoNT/A intoxication. M17 cells were transfected with an expression plasmid for the YFP/SNAP25/CFP fusion protein, a SNAP25 cleavage indicator protein in which SNAP25 is flanked by two different fluorescent proteins, YFP and CFP. The M17 cells were co-transfected with a control plasmid (vector alone), an expression plasmid for ALcB8-TrCP (B8-TrCP) or an expression plasmid for ALcB8 lacking an F-box domain (B8). 24 hrs post-transfection, cells were intoxicated by exposure to 10 nM BoNT/A (+) or left untreated (−). Cell extracts were prepared after 24 hrs of intoxication and the cleavage of indicator by BoNT/A was detected by Western blot with anti-GFP antibody. The presence of undigested indicator protein following BoNT/A exposure results from partial intoxication of M17 cells. From prior studies, the intoxication efficiency of these cells based on endogenous SNAP25 cleavage typically varies between 50% and 80%. Results from three separate representative experiments (out of ten) are shown. (B) Western blot of B8-TrCP after various times of treatment with MG132. M17 cells were transfected with expression plasmid ALcB8 (B8) or ALcB8-TrCP (B8-TrCP) for 24 hrs. Cells were then treated with 10 µM of MG132 for the indicated time before cell lysates were prepared. The expression of ALcB8-TrCP was detected by Western blot with anti-GFP antibody. Unmodified ALcB8-TrCP protein became much more apparent within transfected cells following 4 or 16 hrs of exposure to MG132 and high molecular weight staining proteins accumulate. This implies that ALcB8-TrCP TFB protein is being expressed to a significant extent but undergoes rapid proteasome-mediated turnover resulting in very low steady-state levels. Arrow indicates the unmodified ALcB8-TrCP fusion protein.
(TIF)

Figure S2 **Intracellular localization of ALcB8 TFBs containing variable amounts of TrCP.** M17 cells were transfected with expression plasmids (as indicated) for the various TFB proteins targeting ALc (B8-TrCP and B8-TrCP truncations) or BLc (D5-B10) diagrammed in Figure 1A. Fluorescence microscopy images were taken 24 hrs post-transfection to visualize the YFP fusion partner on each TFB and the images shown are representative of 3 separate experiments.
(TIF)

Figure S3 **TFBs with various TrCP truncations retain activity to protect SNAP25 indicator protein from cleavage by BoNT/A within intoxicated M17 cells.** M17 cells were co-transfected with an expression plasmid for the SNAP25 indicator protein and a second expression plasmid for the indicated TFB protein (diagrams in Figure 1A) or control (vector alone). 24 hrs post transfection, cells in wells were intoxicated by exposure to 10 nM BoNT/A (+) or left unintoxicated (−). Cell extracts were prepared after 24 hrs of intoxication and the extent of cleavage of the indicator by BoNT/A was assessed by Western blots with anti-GFP antibody and the results shown are representative of 3 separate experiments.
(TIF)

Figure S4 **TFB D5-BLcB10 expressed in N2A cells binds to BoNT/B Lc target.** A. TFB D5-BLcB10 (D5-B10) binds to co-expressed GST-BLc in N2A cells based on GST pull-down. Glutathione-transferase (GST) fused to BoNT/B Lc (GST-BLc), or GST (control), each complexed with glutathione magnetic beads, was added to TFB D5-B10 transfected N2A cell extract and the GST proteins were recovered by glutathione affinity. D5-B10 was detected by anti-GFP antibody and shown to be present following GST pull-down of the BLc. B. D5-B10 binds to co-expressed GST-BLc in N2A cells based on streptavidin pull-down. N2A cells were co-transfected with expression plasmids for GFP-BLc and for TFB D5-B10 (fused to streptavidin binding peptide and YFP). The D5-B10 was purified by streptavidin affinity and the pull-down fraction was analyzed for co-purified GFP-BLc by Western blot using anti-GFP antibody. An equivalent aliquot of the unpurified cell extract (input) was also included on the Western blot. Data shown are representative of 3 separate experiments.
(TIF)

Figure S5 **BoNT Lc targeted TFBs reduce the steady-state level of the targeted Lc in neuroblastoma cells expressing both ALc and BLc simultaneously.** N2A cells were co-transfected with expression vectors for both CFP-ALc and CFP-BLc along with another expression plasmid for either the TFB D5-B8 or D5-B10 as indicated. 24 hrs post-transfection, cell extracts were prepared and resolved by SDS-PAGE. CFP-Lc and SNAP25 expression levels were detected by Western blotting using BoNT serotype-specific anti-Lc antisera (ALc or BLc) or anti-SNAP25 antibody. The data shown is representative of 2 separate experiments.
(TIF)

Figure S6 **ALc turnover is accelerated by co-expression with TFB D5-B8.** Expression plasmid for CFP-ALc was transfected into cells stably expressing D5-B8 (ALc TFB) or the control, D5-B10 (BLc TFB), as indicated. Cell lysates were prepared at the indicated time points and resolved by SDS-PAGE. The expression level of CFP-ALc, TFB and SNAP25 were assessed by Western blotting using anti-ALc Ab, anti-GFP Ab or anti-SNAP25 Ab for detection and the data shown are representative of 3 separate experiments.
(TIF)

Figure S7 **TFB-mediated acceleration of BoNT Lc turnover is proteasome-dependent.** N2A cells were co-transfected with expression plasmids for CFP-ALc or CFP-BLc and expression plasmids for TFBs D5-B8 or D5-B10 as indicated. 24 hrs post-transfection, cells were treated with either 10 µM MG132 or a DMSO control. 4 hrs later, cell lysates were prepared and resolved by SDS-PAGE. CFP-Lc expression levels were detected by Western blotting using BoNT serotype-specific anti-Lc antisera and the data shown is representative of 4 separate experiments.
(TIF)

Figure S8 **TFB targeting ALc protects cells from cleavage of endogenous SNAP25 following BoNT/A intoxication.** M17 control cells or M17 cells stably expressing ALc TFB (D5-B8) or BLc TFB (D5-B10) were intoxicated with 10 nM BoNT/A for 5 hrs. Cell lysates were prepared and resolved by SDS-PAGE. The level of SNAP25 cleavage was detected by Western blotting using anti-SNAP25 Ab (A) and the % cleavage was quantified by scanning densitometry (B). Data are presented as the averages ± standard deviation calculated from three experiments and compared by ANOVA. The differences between ALc TFB line and BLc TFB line, and between ALc TFB line and M17, are highly significant (p<0.005).
(TIF)

Figure S9 **ALc TFB (D5-B8) expression promotes accelerated recovery of intact endogenous SNAP25 following BoNT/A intoxication.** Control M17 cells or M17 cells stably expressing ALc TFB D5-B8 or BLc TFB D5-B10 were exposed to 10 nM of BoNT/A for 24 hrs. Cell lysates were prepared at times indicated post-intoxication and resolved by SDS-PAGE. SNAP25 was detected by Western blot using anti-SNAP25 Ab and the data shown are representative of ten separate experiments.
(TIF)
