# Supplementary figures and images for: Accelerated Neuronal Cell Recovery from Botulinum Neurotoxin Intoxication by Targeted Ubiquitination
Source: PLoS One. 2011 May 24;6(5):e20352. doi: 10.1371/journal.pone.0020352 (PMC3101245; doi:10.1371/journal.pone.0020352)

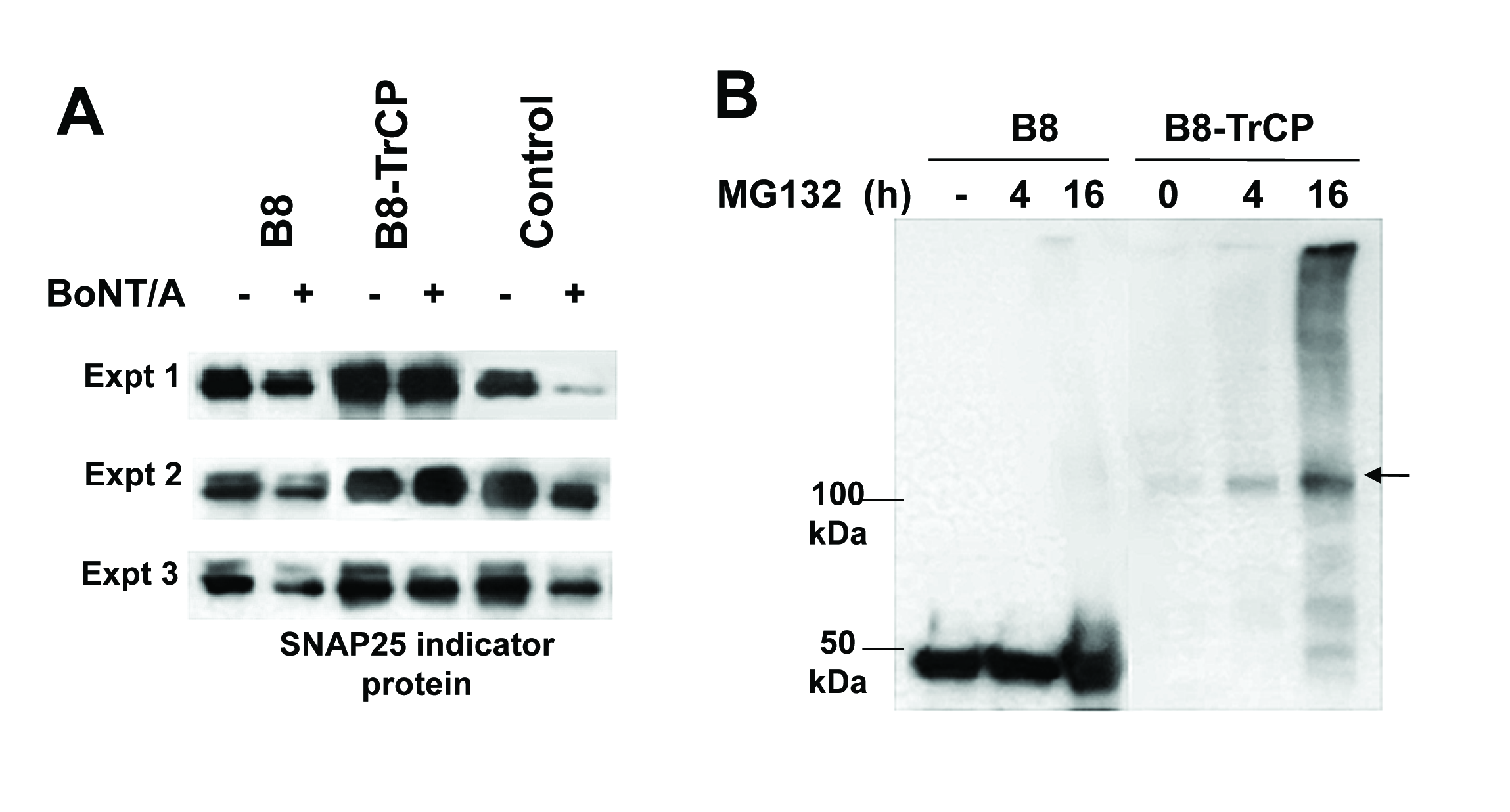

Supplement: Figure S1 — TFB ALcB8-TrCP expressed in M17 cells inhibits BoNT/A cleavage of a SNAP25 indicator protein and is rapidly turned over by a proteasome-mediated process. (A) Western blots of SNAP25 indicator protein levels +/− BoNT/A intoxication. M17 cells were transfected with an expression plasmid for the YFP/SNAP25/CFP fusion protein, a SNAP25 cleavage indicator protein in which SNAP25 is flanked by two different fluorescent proteins, YFP and CFP. The M17 cells were co-transfected with a control plasmid (vector alone), an expression plasmid for ALcB8-TrCP (B8-TrCP) or an expression plasmid for ALcB8 lacking an F-box domain (B8). 24 hrs post-transfection, cells were intoxicated by exposure to 10 nM BoNT/A (+) or left untreated (−). Cell extracts were prepared after 24 hrs of intoxication and the cleavage of indicator by BoNT/A was detected by Western blot with anti-GFP antibody. The presence of undigested indicator protein following BoNT/A exposure results from partial intoxication of M17 cells. From prior studies, the intoxication efficiency of these cells based on endogenous SNAP25 cleavage typically varies between 50% and 80%. Results from three separate representative experiments (out of ten) are shown. (B) Western blot of B8-TrCP after various times of treatment with MG132. M17 cells were transfected with expression plasmid ALcB8 (B8) or ALcB8-TrCP (B8-TrCP) for 24 hrs. Cells were then treated with 10 µM of MG132 for the indicated time before cell lysates were prepared. The expression of ALcB8-TrCP was detected by Western blot with anti-GFP antibody. Unmodified ALcB8-TrCP protein became much more apparent within transfected cells following 4 or 16 hrs of exposure to MG132 and high molecular weight staining proteins accumulate. This implies that ALcB8-TrCP TFB protein is being expressed to a significant extent but undergoes rapid proteasome-mediated turnover resulting in very low steady-state levels. Arrow indicates the unmodified ALcB8-TrCP fusion protein. (TIF) [file pone.0020352.s001.tif]

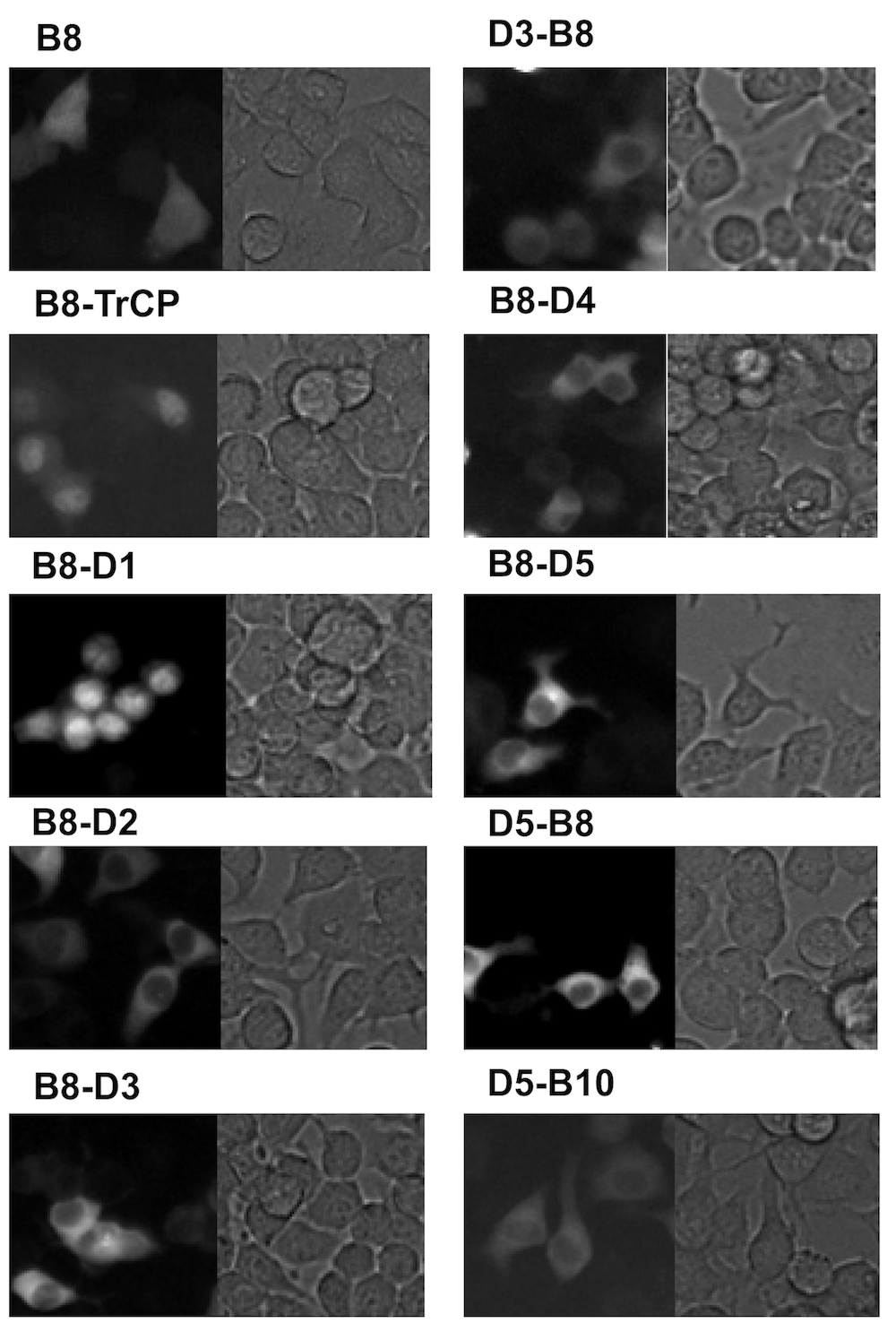

Supplement: Figure S2 — Intracellular localization of ALcB8 TFBs containing variable amounts of TrCP. M17 cells were transfected with expression plasmids (as indicated) for the various TFB proteins targeting ALc (B8-TrCP and B8-TrCP truncations) or BLc (D5-B10) diagrammed in Figure 1A. Fluorescence microscopy images were taken 24 hrs post-transfection to visualize the YFP fusion partner on each TFB and the images shown are representative of 3 separate experiments. (TIF) [file pone.0020352.s002.tif]

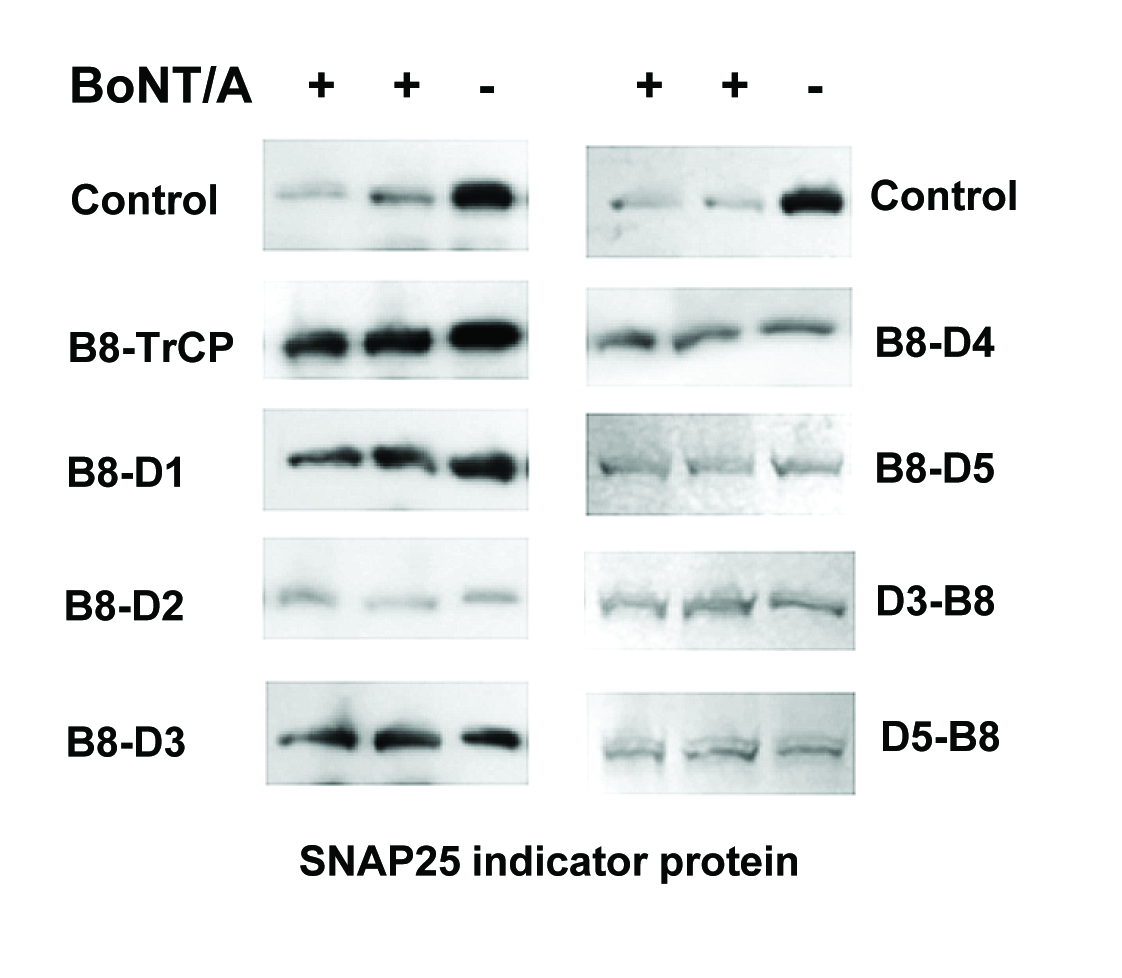

Supplement: Figure S3 — TFBs with various TrCP truncations retain activity to protect SNAP25 indicator protein from cleavage by BoNT/A within intoxicated M17 cells. M17 cells were co-transfected with an expression plasmid for the SNAP25 indicator protein and a second expression plasmid for the indicated TFB protein (diagrams in Figure 1A) or control (vector alone). 24 hrs post transfection, cells in wells were intoxicated by exposure to 10 nM BoNT/A (+) or left unintoxicated (−). Cell extracts were prepared after 24 hrs of intoxication and the extent of cleavage of the indicator by BoNT/A was assessed by Western blots with anti-GFP antibody and the results shown are representative of 3 separate experiments. (TIF) [file pone.0020352.s003.tif]

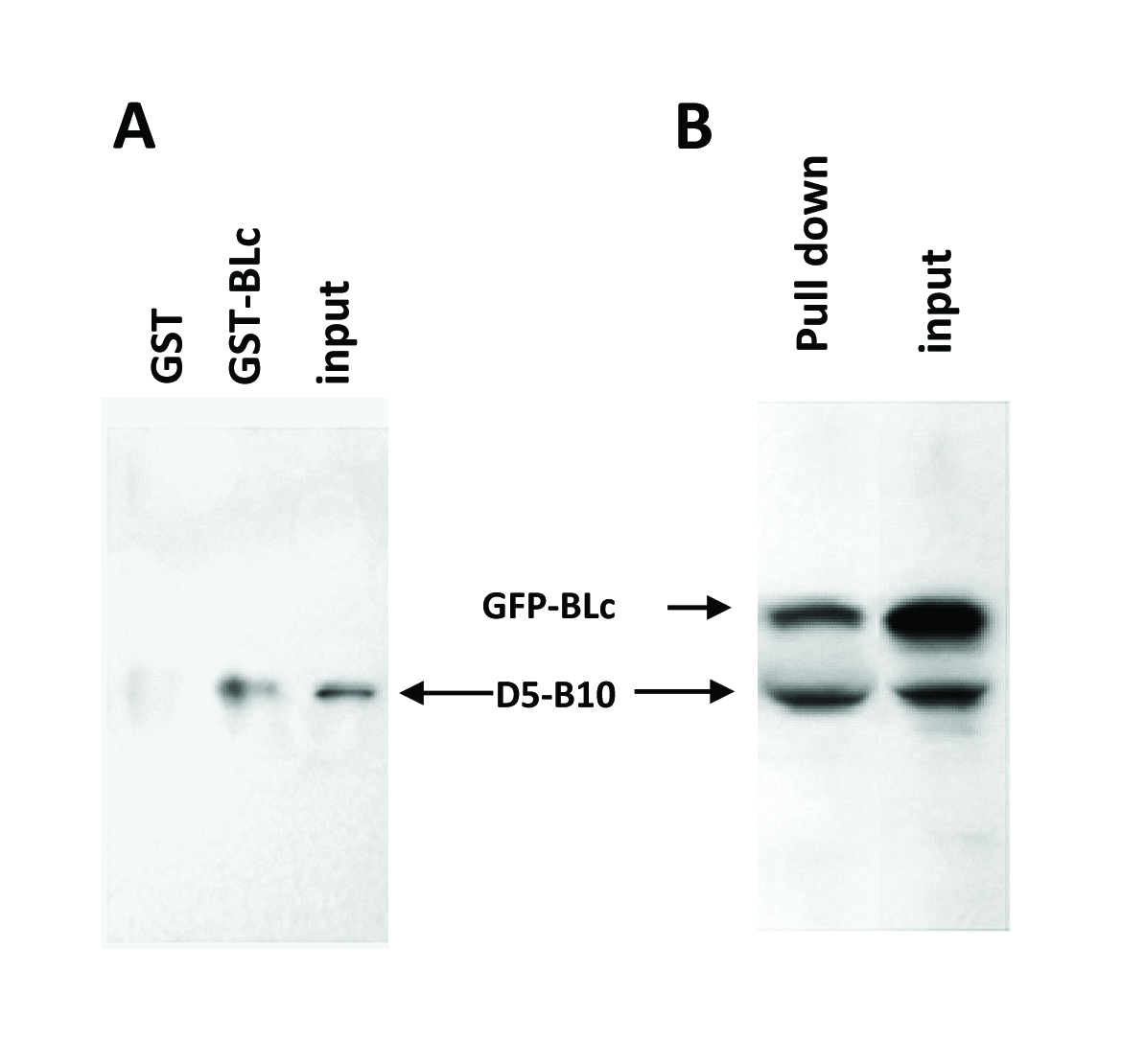

Supplement: Figure S4 — TFB D5-BLcB10 expressed in N2A cells binds to BoNT/B Lc target. A. TFB D5-BLcB10 (D5-B10) binds to co-expressed GST-BLc in N2A cells based on GST pull-down. Glutathione-transferase (GST) fused to BoNT/B Lc (GST-BLc), or GST (control), each complexed with glutathione magnetic beads, was added to TFB D5-B10 transfected N2A cell extract and the GST proteins were recovered by glutathione affinity. D5-B10 was detected by anti-GFP antibody and shown to be present following GST pull-down of the BLc. B. D5-B10 binds to co-expressed GST-BLc in N2A cells based on streptavidin pull-down. N2A cells were co-transfected with expression plasmids for GFP-BLc and for TFB D5-B10 (fused to streptavidin binding peptide and YFP). The D5-B10 was purified by streptavidin affinity and the pull-down fraction was analyzed for co-purified GFP-BLc by Western blot using anti-GFP antibody. An equivalent aliquot of the unpurified cell extract (input) was also included on the Western blot. Data shown are representative of 3 separate experiments. (TIF) [file pone.0020352.s004.tif]

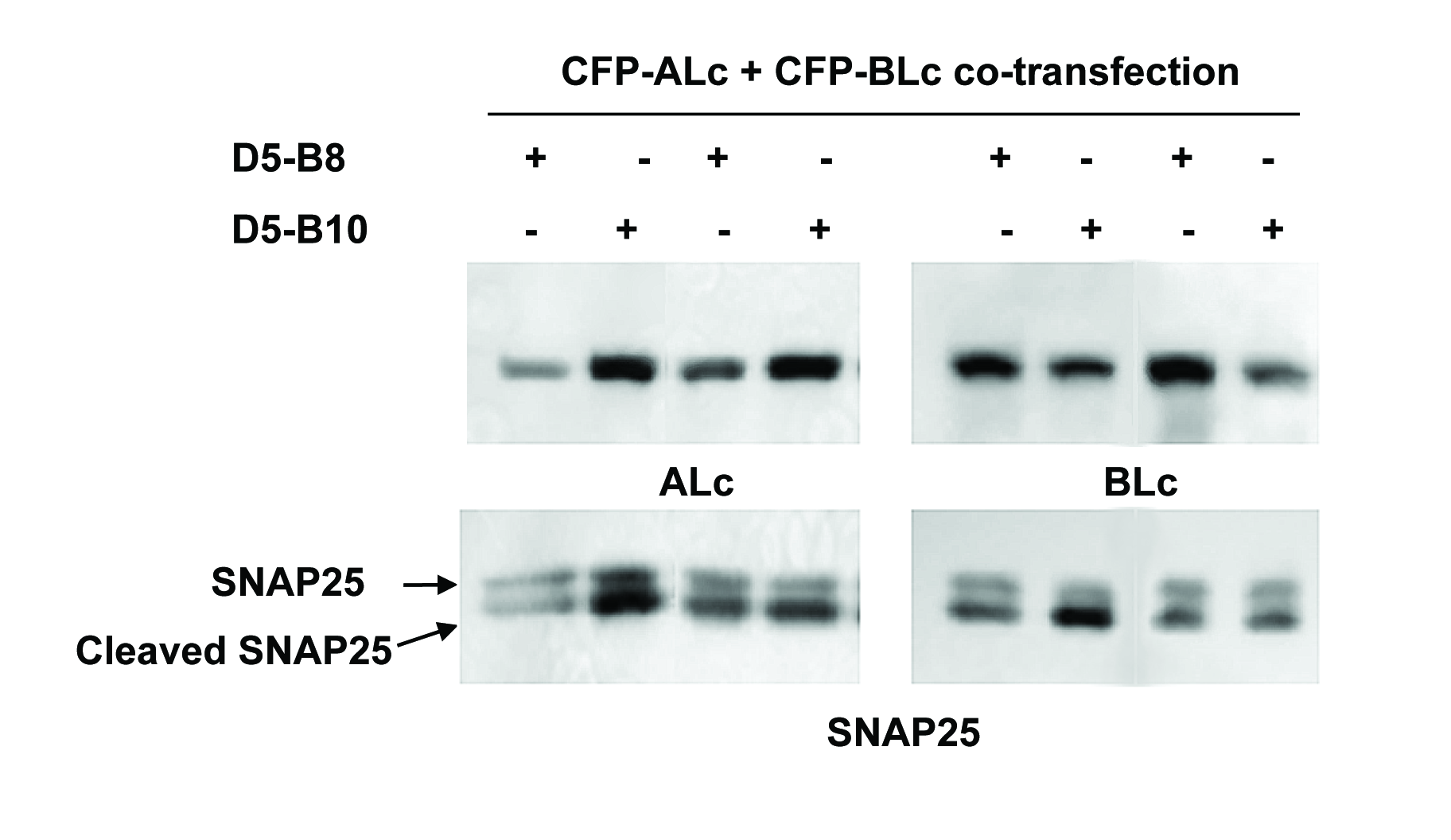

Supplement: Figure S5 — BoNT Lc targeted TFBs reduce the steady-state level of the targeted Lc in neuroblastoma cells expressing both ALc and BLc simultaneously. N2A cells were co-transfected with expression vectors for both CFP-ALc and CFP-BLc along with another expression plasmid for either the TFB D5-B8 or D5-B10 as indicated. 24 hrs post-transfection, cell extracts were prepared and resolved by SDS-PAGE. CFP-Lc and SNAP25 expression levels were detected by Western blotting using BoNT serotype-specific anti-Lc antisera (ALc or BLc) or anti-SNAP25 antibody. The data shown is representative of 2 separate experiments. (TIF) [file pone.0020352.s005.tif]

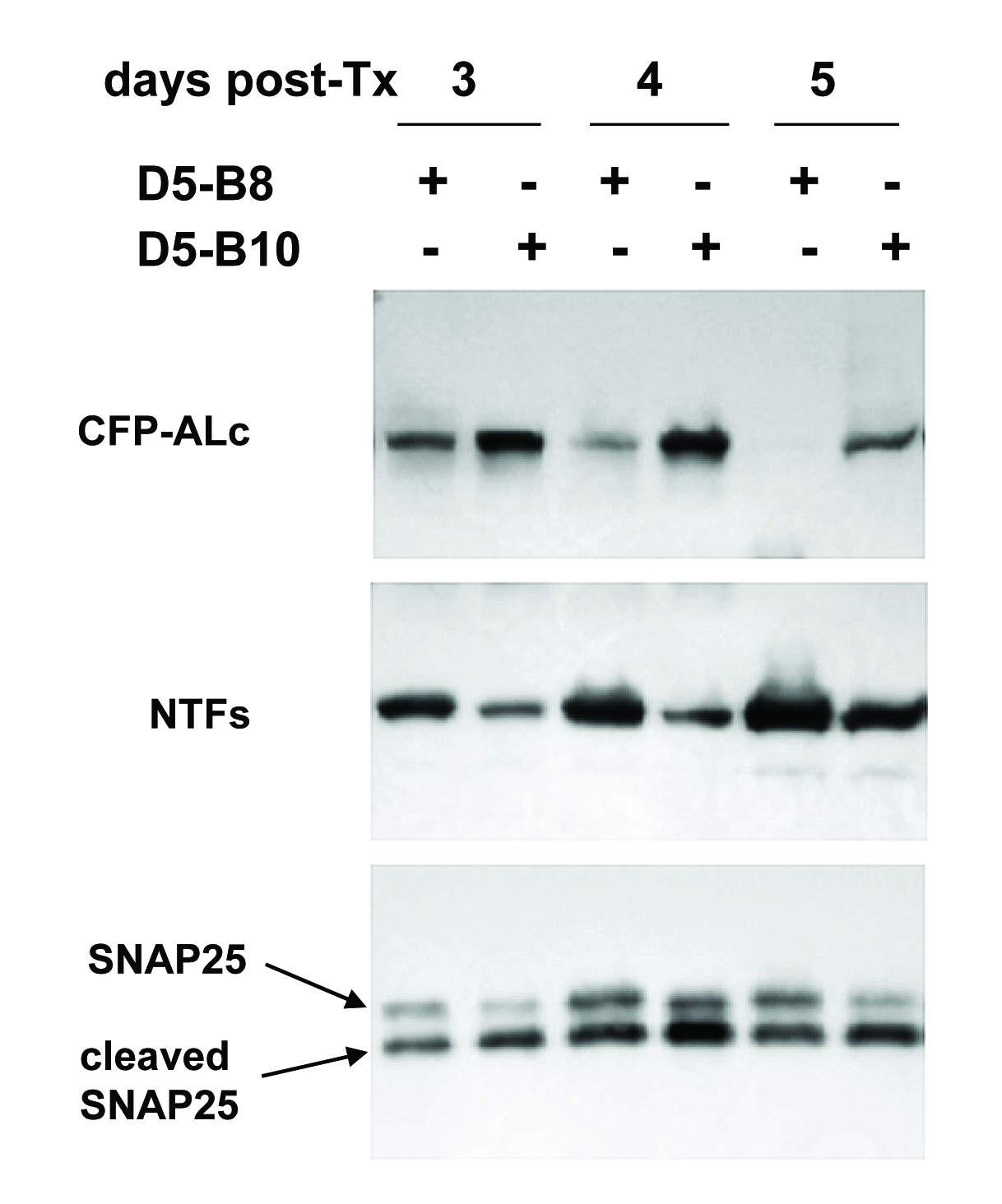

Supplement: Figure S6 — ALc turnover is accelerated by co-expression with TFB D5-B8. Expression plasmid for CFP-ALc was transfected into cells stably expressing D5-B8 (ALc TFB) or the control, D5-B10 (BLc TFB), as indicated. Cell lysates were prepared at the indicated time points and resolved by SDS-PAGE. The expression level of CFP-ALc, TFB and SNAP25 were assessed by Western blotting using anti-ALc Ab, anti-GFP Ab or anti-SNAP25 Ab for detection and the data shown are representative of 3 separate experiments. (TIF) [file pone.0020352.s006.tif]

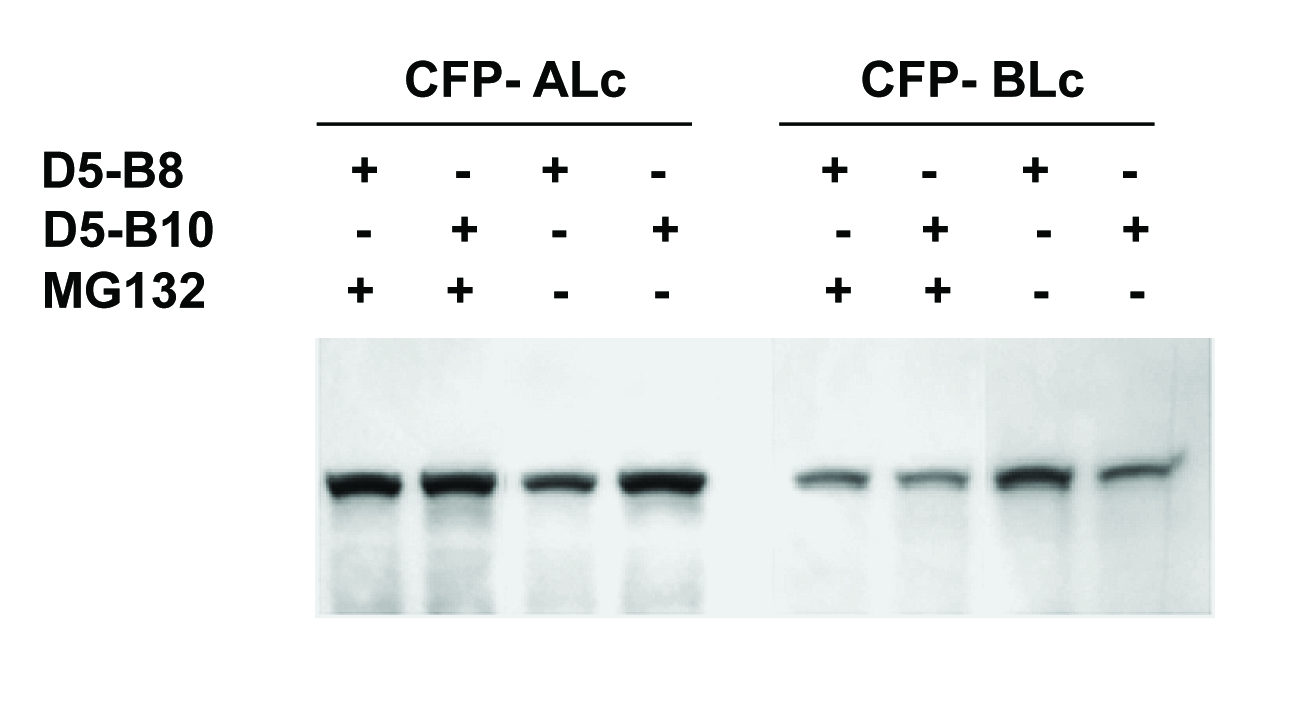

Supplement: Figure S7 — TFB-mediated acceleration of BoNT Lc turnover is proteasome-dependent. N2A cells were co-transfected with expression plasmids for CFP-ALc or CFP-BLc and expression plasmids for TFBs D5-B8 or D5-B10 as indicated. 24 hrs post-transfection, cells were treated with either 10 µM MG132 or a DMSO control. 4 hrs later, cell lysates were prepared and resolved by SDS-PAGE. CFP-Lc expression levels were detected by Western blotting using BoNT serotype-specific anti-Lc antisera and the data shown is representative of 4 separate experiments. (TIF) [file pone.0020352.s007.tif]

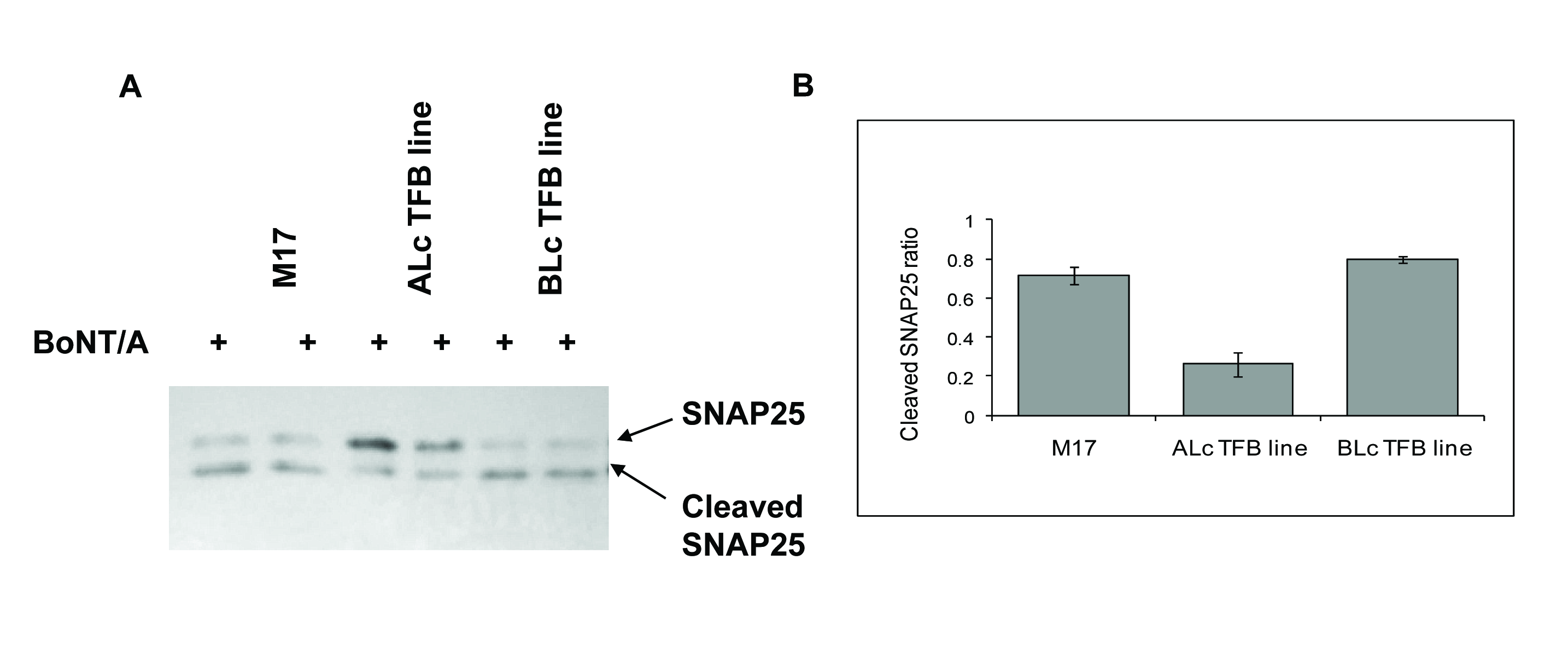

Supplement: Figure S8 — TFB targeting ALc protects cells from cleavage of endogenous SNAP25 following BoNT/A intoxication. M17 control cells or M17 cells stably expressing ALc TFB (D5-B8) or BLc TFB (D5-B10) were intoxicated with 10 nM BoNT/A for 5 hrs. Cell lysates were prepared and resolved by SDS-PAGE. The level of SNAP25 cleavage was detected by Western blotting using anti-SNAP25 Ab (A) and the % cleavage was quantified by scanning densitometry (B). Data are presented as the averages ± standard deviation calculated from three experiments and compared by ANOVA. The differences between ALc TFB line and BLc TFB line, and between ALc TFB line and M17, are highly significant (p<0.005). (TIF) [file pone.0020352.s008.tif]

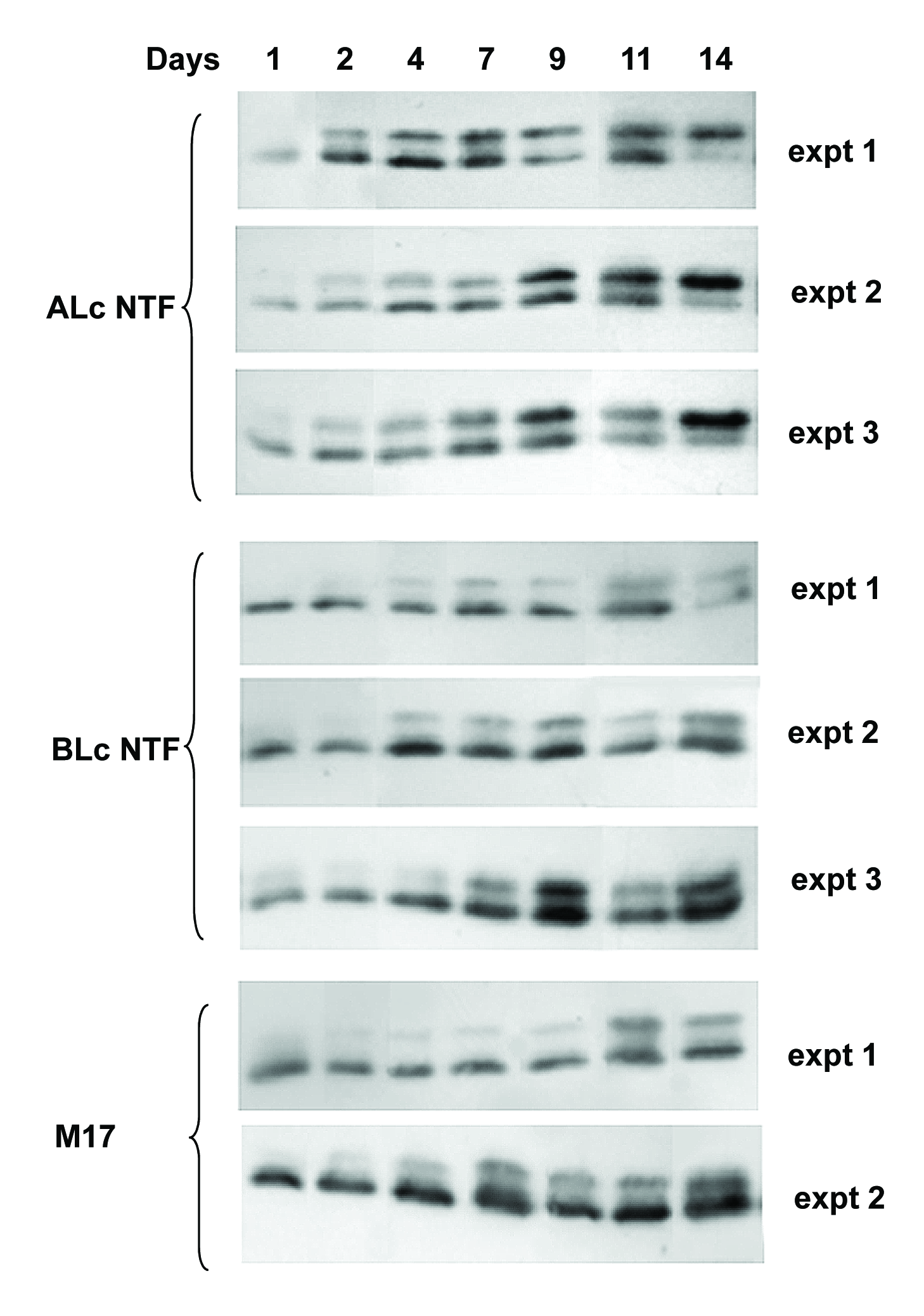

Supplement: Figure S9 — ALc TFB (D5-B8) expression promotes accelerated recovery of intact endogenous SNAP25 following BoNT/A intoxication. Control M17 cells or M17 cells stably expressing ALc TFB D5-B8 or BLc TFB D5-B10 were exposed to 10 nM of BoNT/A for 24 hrs. Cell lysates were prepared at times indicated post-intoxication and resolved by SDS-PAGE. SNAP25 was detected by Western blot using anti-SNAP25 Ab and the data shown are representative of ten separate experiments. (TIF) [file pone.0020352.s009.tif]
